# Evidence Linking Brain Activity Modulation to Age and to Deductive Training

**DOI:** 10.1155/2018/1401579

**Published:** 2018-11-25

**Authors:** P. Álvarez Merino, C. Requena, F. Salto

**Affiliations:** Department of Psychology, Sociology and Philosophy, University of León, Campus de Vegazana, s/n, 24071 León, Spain

## Abstract

Electrical brain activity modulation in terms of changes in its intensity and spatial distribution is a function of age and task demand. However, the dynamics of brain modulation is unknown when it depends on external factors such as training. The aim of this research is to verify the effect of deductive reasoning training on the modulation in the brain activity of healthy younger and older adults (*N* = 47 (mean age of 21 ± 3.39) and *N* = 38 (mean age of 68.92 ± 5.72)). The analysis reveals the benefits of training, showing that it lowers cerebral activation while increasing the number of correct responses in the trained reasoning task (*p* < 0.001). The brain source generators were identified by time-averaging low-resolution brain electromagnetic tomography (sLORETA) current density images. In both groups, a bilateral overactivation associated with the task and not with age was identified. However, while the profile of bilateral activation in younger adults was symmetrical in anterior areas, in the older ones, the profile was located asymmetrically in anterior and posterior areas. Consequently, bilaterality may be a marker of how the brain adapts to maintain cognitive function in demanding tasks in both age groups. However, the differential bilateral locations across age groups indicate that the tendency to brain modulation is determined by age.

## 1. Introduction

Brain activity adapts in time to the cognitive needs of an organism, altering its intensity and its distribution which can be measured through modulations in electrical brain activity [1]. Scientific literature offers two different approaches to the complex phenomenon of the electrical brain signal's modulation. One explicative strategy focuses on overactivation of brain activity as a function of the demand of tasks [2], while a second perspective explains modulation in terms of age and focuses on the idiosyncrasy of brain aging [3]. Many studies have verified how brain overactivation correlates with better performance in cognitive tasks. For example, the performance of older people who participated in a digit span task was better if they exhibited a bilateral pattern of brain activity compared to those who did not show such bilaterality [4]. Another memory study involving older adults with high and low memory capacities and younger adults reported that both the older adults with low memory capacity and the younger adults showed prefrontal asymmetry with greater activity in the right hemisphere [3]. While the performance of the younger adults was the most successful, that of the older adults with low memory capacity was the least. In the older adults with better memory, a pattern of bilateral activity was evidenced with similar results to those of the younger adults. When the task is more demanding for these older adults, the left prefrontal cortex is also activated. That is, a supplementary activation reflects the additional effort that these older adults make in order to access information [5]. On the other hand, the approach based on the difficulty gradient of the task describes the increment in brain activation and the involvement of wider brain areas as an adaptive strategy for functional performance both in older and in younger adults [6]. In this regard, an investigation in which younger and older adults had to resolve memory tasks of different complexity levels found that the dorsolateral prefrontal cortex was overactivated in older adults in order to achieve a performance similar to that of younger adults. In addition, as the difficulty increased, the dorsolateral prefrontal cortex in younger adults activated as well [7].

The second perspective attributes brain activity modulation to age [8]. Namely, the cerebral plastic behavior in older adults makes it possible for them to relearn a new activation mode which is manifested in a deactivation of posterior regions along with a higher activation in previous regions [9]. For example, in tasks requiring the intervention of basic cognitive operations such as visual perception, the highest activation is not located in the posterior cognitive regions of older adults. This brain behavior is associated with age, but it does not necessarily involve cognitive decline [10]. In this regard, an extensive research study tested age-related cerebral changes using an episodic memory task (high complexity) as well as a visual perception one (low complexity) [11]. The results showed that regardless of the complexity of the task, a higher activation was localized in the prefrontal cortex. In addition, this activation correlates with better cognitive function, as opposed to an inverse correlation between performance and activity in the occipital region. Other studies have also shown age-related changes in the brain and cognitive strategies to solve executive control tasks [12]. In particular, the experiment was carried out with younger and older adults who faced a task with consecutive pairs whereby the first one constituted a cue and the second one a target. Subjects were instructed to respond to the target whenever it was preceded by the same cue. Otherwise, they should omit or refrain from responding. The results showed that, with age, the executive control strategies shifted in temporal distribution. That is, they were proactive in younger adults during the presentation of the cue and reactive in older adults in response to the target presentation. In addition, the neuroimaging analysis showed that only younger adults displayed higher activation of the dorsolateral prefrontal cortex and left hippocampus when the cue appeared rather than when the target was presented. On the other hand, it reveals an idiosyncratic behavior of cerebral aging in the amount of cognitive resources that are activated by the demand of a cognitive task. There seems to be a ceiling effect linked to how high the difficulty of the demand is in older adults, since brain activity lessens if the task is too hard [13]. An investigation in which younger and older adults had to resolve memory tasks of different complexity levels found that the prefrontal cortex was overactivated in both younger and older adults to achieve performance success [7]. However, beyond a certain level of demand, in older adults as opposed to younger adults, brain activity decreased, and so did their performance [14].

All in all, there is evidence of both task-dependent and age-dependent factors in brain activity modulation. Demanding tasks modulate brain activity both in younger and in older adults. However, simultaneously beyond the task demand, there is also idiosyncratic aged brain behavior. The state of the art includes two basic results: (1) when older adults face a demanding task, they require more brain activation than younger adults to obtain a similar performance level [6] and (2) when the task is demanding for all age groups, younger adult brains manifest an increased activation while older adult brains tend to diminish their brain activity [15, 16]. However, there are no known experimental studies that analyze the effect of training on the modulation of brain activity which in turn would help to clarify the relationship between the two explanatory approaches to brain modulation.

The current study is aimed at examining the modulation of EEG brain activity in a highly demanding cognitive task in younger and older adults before and after a reasoning training. In particular, the following hypotheses were tested experimentally: (a) baseline EEG activity will show a bilateral overactivation in younger adults rather than in older ones, (b) the posttraining evaluation will show bilateral overactivation in the older adults while it will disappear in the younger ones, and (c) the effect of training improves deductive reasoning performance by increasing the number of valid responses and decreasing the reaction time in both age groups.

## 2. Materials and Methods

### 2.1. Participants

Eighty-five subjects, divided into two age groups, voluntarily participated in this study. The group of younger adults consisted of 47 subjects with an average age of 24.21 ± 3.39 years. These subjects were students from the University of León whose participation was rewarded with 1 academic credit. On the other hand, the group of older adults consisted of another 38 subjects with an average age of 68.92 ± 5.72 years. These subjects were contacted through the senior center of León Council.

All participants were screened to be right-handed with normal or corrected vision and not currently under any stress (i.e., exams, job interviews, and grief). Additionally, older adults were screened to be cognitively intact (Mini-Mental Status Exam ≥ 28) [17]. This study was approved by the Ethics Committee of the University of León in 2017, and it was carried out following the deontological standards recognized by the Helsinki Declaration of 1975 (as revised in the 52nd Annual General Assembly in Edinburgh, Scotland, in October 2000), the standards of Good Clinical Practice, and the Spanish Legal Code regulating clinical research involving human subjects (Royal Decree 223/2004 about regulation of clinical trials).

### 2.2. Procedure

The experimental design consists of 99 deductive reasoning tasks that are presented in a time window of 3.5 seconds which includes the presentation of the task and the response time. There was an interval of 200 ms between tasks. Tasks were presented through the Mind Tracer (Neuronic S.A., Havana) on a 23-inch NEC screen. Subjects were requested to minimize their blinking as well as their postural movements. The program also provides conductual information about the number of correct and incorrect answers and reaction times.

For the basal evaluation and for posttraining evaluation, 99 deductive tasks were designed corresponding to the three types presented above. 33 items for each type of tasks were randomly distributed. In the basal evaluation, subjects were instructed to mandatorily respond to this instruction: “If the item follows a rule based on properties regarding figures, colors, number, shape, or shading, press the ‘Ctrl' key; otherwise, press the ‘spacebar' key.” Notice that in the basal evaluation, the subject had no information or hints about the contents of the rules. The task was new and highly demanding, since the subject lacked any hints and did not know the rules of the task. For the posttraining, the subjects followed this instruction: “If the three cards have two or more properties in common, then (and only then) they form a set.” In this case, subjects would respond by pressing the “Ctrl” key and otherwise by pressing the spacebar.

The initial registration lasted about 40 minutes: 20 minutes to prepare the EEG system (cleaning, placement of the electrodes, etc.) using MEDICID (Neuronic S.A., Havana) and another 20 minutes to run the test. After the initial recording, subjects undertook behavioural training of reasoning in a single session followed by a posttraining recording, with an approximate duration of 70 minutes: 30 minutes for the training itself and the remaining time for EEG data recording.

### 2.3. Stimuli

The paradigm used during the EEG acquisition was based on a subset of the cards that compose the deck of the card game set. The game was unknown to all participants, and it was instrumental in the training and evaluation of elemental logical deductions. This kind of task was chosen because human deductive abilities are known to be, under certain specific conditions, rather invariant with respect to age, culture, and education. In particular, the reduction of cognitive resources accompanying aging does not impede the preservation of elementary deductive abilities [18], specifically if inferential conclusions are relevant to their premises [17, 19]. Even if the cultural context partially determines reasoning, elementary deductive inference remains invariant across cultures [20] and education levels [21]. Additionally, deductive inferences occur both in linguistic and in visual support [22]. That is, there are deductions which are not sentential sequences of premises and conclusions but logically valid visual inferences such as those present in diagrams or geometrical proofs. Finally, an interesting peculiarity of deductive reasoning is its easiness to produce new tasks with simple instructions where it is easy to measure and control both the logical complexity (number of instances of employed rules) and the relational complexity (number of variables). In this research, these measures offer an objective demand gradient.

The deductive tasks presented and evaluated in this study are elementary logical (first order) inferences realized over a subset of the card game set. The cards presented items with certain characteristics: shape, color, number, and shading. There are three shapes, two colors, two numbers, and two shadings. Each item presents a trio of cards which shares zero, one, two, or three of these characteristics. By definition, any trio is a set if and only if the three cards share at least two properties. Determining whether an item (trio) is or is not a set is a purely deductive task, namely, a finite sequence of inferences which can be developed in a logically valid way and follows a recursive procedure which computes the truth values of the premises. Given that the subject perceives the properties of each card, the exercise of computing or deducing if the trio is a set is an elementary logical task. In the simplest scenario, it is enough to apply the definition of a set to verify that in fact, the cards in the trio share two properties. This situation automatically applies the rule of modus ponens (deduce B from {A, if A then B}). In the more complex scenario, instead of directly applying the definition after positive cases, we have counterexamples. In this case, the rule of modus tollens (deduce notA from {notB, if A then B}) can be used.

**Figure 1:**
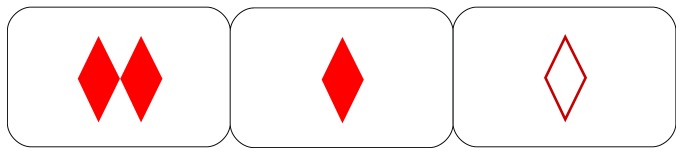
Case 1 type.

To elucidate the experimental design, we show an example of each of the three kinds of items presented to the subjects in the evaluation tests. Only in the first kind of case (see Figure 1) do the three cards share at least two properties, and consequently the trio satisfies the definition of a set.

The three cards in Figure 1 share the same shape and color; therefore, they are a set. From the only presentation of the trio and the application of negationless deductive rules, the reasoner may deduce that the item is a set. Observe how the reasoner may deduce the conclusion without going through all the properties of all objects in the cards: once form and color are shared, there is no need for further verifications.

**Figure 2:**
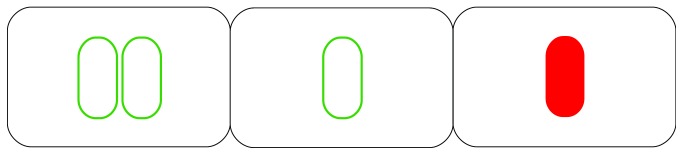
Case 2 type.

The second type of case is a trio which shares only one property (see Figure 2).

The three cards share shape, but no other property. Therefore, this item is not a set. The inference behind this conclusion contains an application of the modus tollens rule, since by this rule the subject can refute each of the other properties (color, number, and shading) one by one.

**Figure 3:**
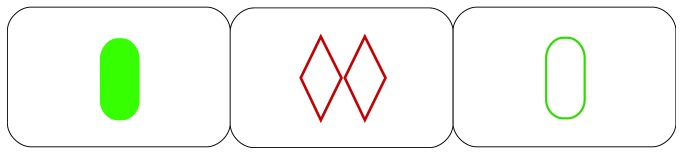
Case type 3.

Finally, Figure 3 shows the example of the third type of case in which no properties are shared by any card of the trio.

The subject may easily verify property by property that a characteristic is not shared in the trio, deducing by modus tollens that the trio is not a set. Note that in the three types of cases, the whole inference is elementary and deductively valid. Moreover, it is remarkable that the second and third types of cases have a slightly greater logical complexity than case 1, but their relational complexity is identical.

### 2.4. Training

All subjects received a personalized one-on-one training, which took place in the same session as the postevaluation. The point of the training was to produce valid deductive inferences by means of recursive or computable logical procedures which would prove whether a given item was (or was not) part of a set. In its first stage, the training focused on identifying sets and practicing at least 15 exercises of type 1 tasks with items that were not present in the basal evaluation. In its second stage, the training made the subject explain her deductive process out loud to the researcher who then corrected her as necessary. Since several logically equivalent procedures are equally acceptable, the personalized training adapts to the heuristical strategies proposed by the subject in case they are logically valid. Remarkably, the deductive training does not purport to teach the subject to reason logically but to bring into explicit conscience the logical properties of the inferences she already makes. For example, the conjunction operator (logical operator for “and”) allows the subject to go over several cards to accumulate available conclusions. This initial training phase ends when the subject says she understands the task of identifying positive cases of a set and does not commit two consecutive errors in type 1 trials.

In the third training phase, the subject herself proposed examples of items that would be a set and described her reasoning out loud. Once her proposals were adequate, the training for types 2 and 3 began, which consisted of making the subject aware of the use of modus tollens to infer counterexamples to sets. For example, if one card did not share a property with another one in a given item, it was deduced to be a counterexample to a set. The training finished when the subject expressed her cognizance of the task and did not commit any errors. The standard duration of the personalized training process was between 20 and 30 minutes for each subject.

## 3. EEG Recording and Analysis

The EEG was recorded with a 64-channel amplifier (Neuronic System, Havana) and specific acquisition software (Neuronic EEG/Edition EEG Software). Reference electrodes were placed on the earlobes. In addition, electrooculography (EOG) was registered using three pairs of external electrodes in order to record the horizontal and vertical movement of the eyes. Electrode impedance was set for each subject before data collection but always kept below 5 kΩ. The recording was carried out using an Electrocap with Ag/AgCl electrodes, which made it possible to analyze the active scalp areas of the subjects. ERP signals and stimulus markers were continuously recorded at a sampling frequency of 200 Hz during the 20-minute presentation of the task. The signals were filtered using a band-pass finite impulse response filter with a Hamming window between 1 and 70 Hz. In addition, a 50 Hz notch filter was used in order to remove the power line artifact. Finally, a three-step artifact rejection algorithm was applied to minimize oculographic and myographic artifacts [23]: (1) components related to eye blinks, according to a visual inspection of the scalp maps and their temporal activations from independent component analysis (ICA), were discarded [24], (2) segmentation of each 3.5-second trial into one 1.5 s length trial ranging from 200 ms before stimulus onset to 1300 ms after stimulus onset, and (3) the thresholding of amplitude in each trial was established in five standard deviations of the signal. That is, trials in which at least five channels contained two samples that exceeded the threshold were taken out. Only correct answers were considered for further analysis. Next, a sliding window approach was used for the localization of the major activation of each trial. Windows of 150 ms with an overlap of 90% were selected for measures of brain activity before and after training. Peak amplitude measurement took into account the most negative peak value within the temporal window of 400 to 550 ms after the stimulus.

### 3.1. Source Localization

This technique has been widely used to study the neural correlations of cognition, because it combines a high temporal resolution of the EEG technique with a reasonable spatial identification of the electrical signal of the cortical sources [25] (see http://www.uzh.ch/keyinst/NewLORETA/sLORETA/sLORETA.htm). The sLORETA software divides the brain into a total of 6239 cubic voxels with a resolution of 5 mm and estimates the density of the current sources [26].

In the current investigation, the source localization was estimated with the analysis of 64 electrodes located in the frontal, medial, temporal, and bilateral parietal regions. The subjects were registered using the International 10-20 system. The sources were calculated for every subject and each age group at the temporal window of 400 to 550 ms with the Brain Cracker (Neuronic S.A., Havana) which used the low-resolution electromagnetic tomography (LORETA implemented in sLORETA [26]). sLORETA source current density is calculated from the scalp-recorded ERP using a realistic head model from the Montreal Neurological Institute (MNI) [27], in which the 3-D solution space was restricted to only the cortical gray matter [28]. The ERP voltage topographic maps were made by plotting color-coded isopotentials obtained by interpolating the voltage values between the scalp electrodes in specific latencies. Voxelwise nonparametrical statistics as implemented in sLORETA were used.

## 4. Results

EEG records were processed using the “EEG edition” software (Neuronic S.A., Havana). Descriptive analyses for each group were calculated using a toolbox from MATLAB R2015a, which was developed in the laboratory of the researchers. Statistical analyses were performed using Statistica (Statistica 10). Brain Cracker and sLORETA software were used to determine source localization [26].

### 4.1. Correct versus Incorrect Responses

**Figure 4:**
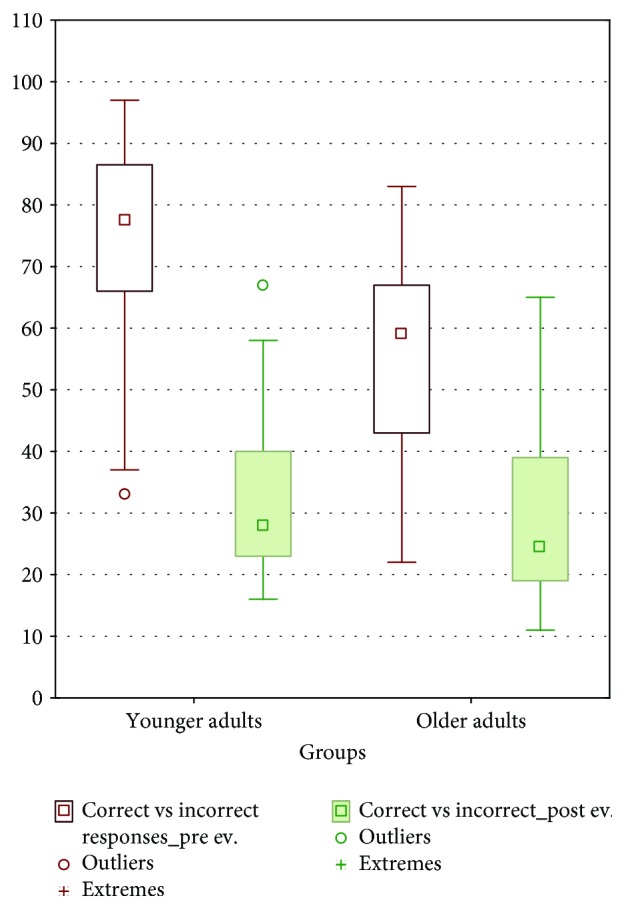
Mean and SD of correct and incorrect responses in younger and older adults.

In relation to the number of correct responses in the basal evaluation, the younger adults obtained an average of 32.08 ± 12.29 whereas the group of older adults obtained an average of 31.05 ± 15.86. Regarding the number of correct response evaluations posttraining, the younger adults obtained an average of 74.79 ± 14.45 whereas the older adults obtained an average of 55.13 ± 16.94 (Figure 4).

To evaluate the effects of this training, a repeated measures model was used. The difference in the number of correct/incorrect responses between both age groups was significant (*F*_1,84_ = 24.186, *p* = 0.001), just like in postevaluation (*F*_1,84_ = 188.596, *p* = 0.001). It shows that the interaction between groups and postevaluation is significant (*F*_1,84_ = 14.674, *p* = 0.001) and the level of confidence was 0.95.

**Figure 5:**
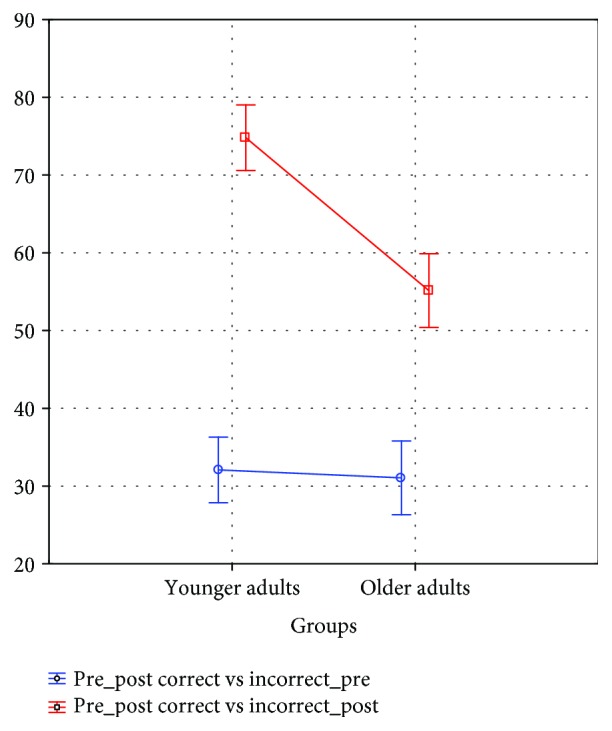
Significant differences between correct/incorrect responses preevaluation and postevaluation, within age groups.

Post hoc analysis using the Tukey test revealed significant differences in the pre-postevaluation, both in the group of younger adults (*p* < 0.001) and in the group of older adults (*p* < 0.001). The training had a greater effect on the group of younger adults (Table 1 and Figure 5).

### 4.2. Reaction Times

**Figure 6:**
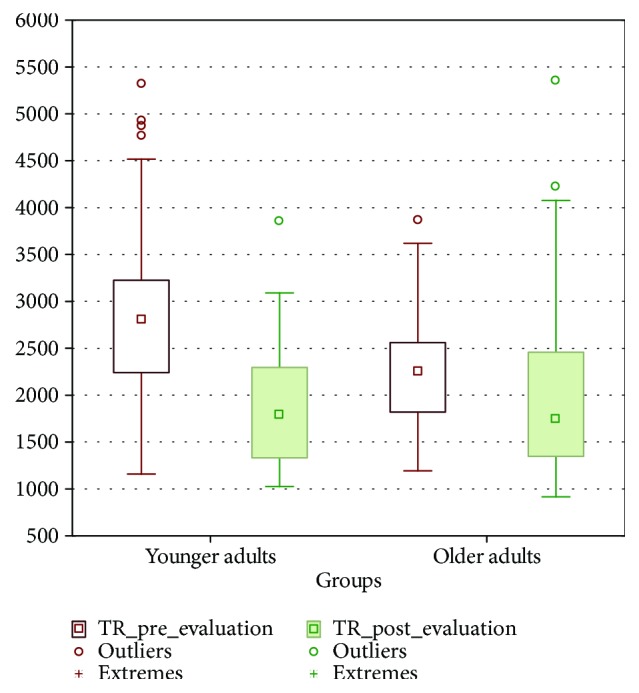
Mean and SD of reaction times in younger and older adults.

The average reaction time in the basal evaluation was 1869.33 ± 608.79 ms for younger adults and 2097.89 ± 1046.77 ms for the older ones. The same parameter posttraining was 2898.50 ± 917.93 ms for younger adults and 2275.42 ± 635.92 ms for older adults (Figure 6).

To evaluate the effects of this training, a repeated measures model was used. The difference in reaction times between both age groups was not significant (*F*_1,84_ = 1.700, *p* = 0.195). However, the posttraining shows significant differences (*F*_1,84_ = 40.920, *p* = 0.001), especially in the group of younger adults. This is verified in the effect of the interaction, which is also significant (*F*_1,84_ = 20.410, *p* = 0.001), because the level of confidence is 0.95.

**Figure 7:**
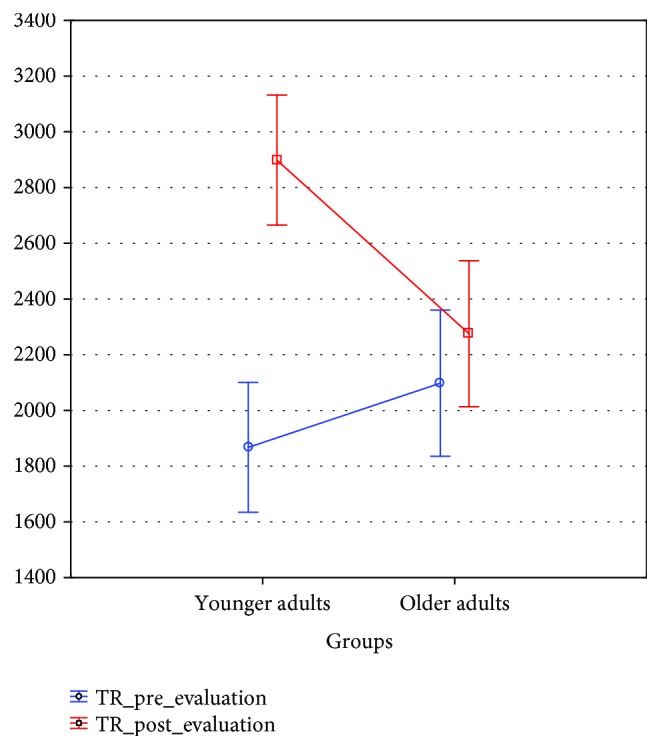
Significant differences between reaction times preevaluation and postevaluation, within age groups.

Post hoc analysis using the Tukey test reveals significant differences in the pre-postevaluation in the group of younger adults (*p* < 0.001) but not in the older adults (*p* < 0.780) (Table 2 and Figure 7).

### 4.3. Analysis of Source Localization with sLORETA

**Figure 8:**
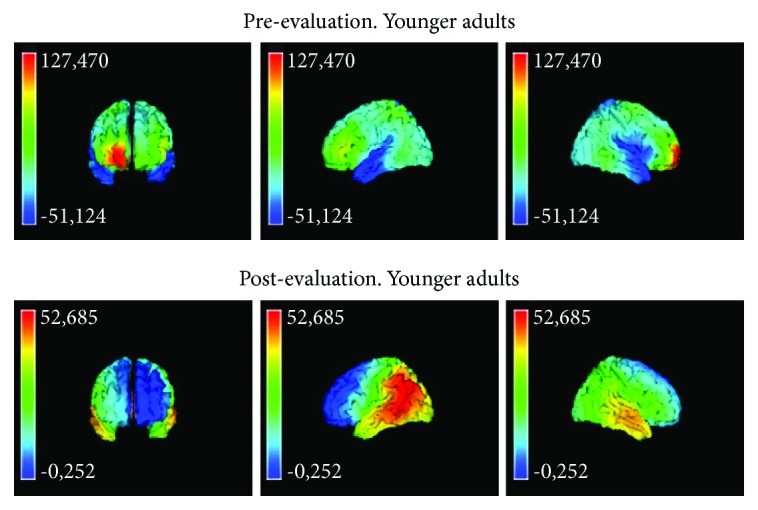
Analysis of source localization pre-postevaluation in younger adults.

The descriptive analyses show that, in the basal evaluation of younger adults, there is activity in the right hemisphere gyrus: orbitofrontal, superior temporal, and postcentral, as well as in the insula; there is also activity in the left superior, middle, and inferior temporal gyri. After training, that is, in the postevaluation of the group of younger adults, the activity was observed in the left hemisphere gyrus: angular, middle temporal, superior, and middle frontal (Figure 8).

**Figure 9:**
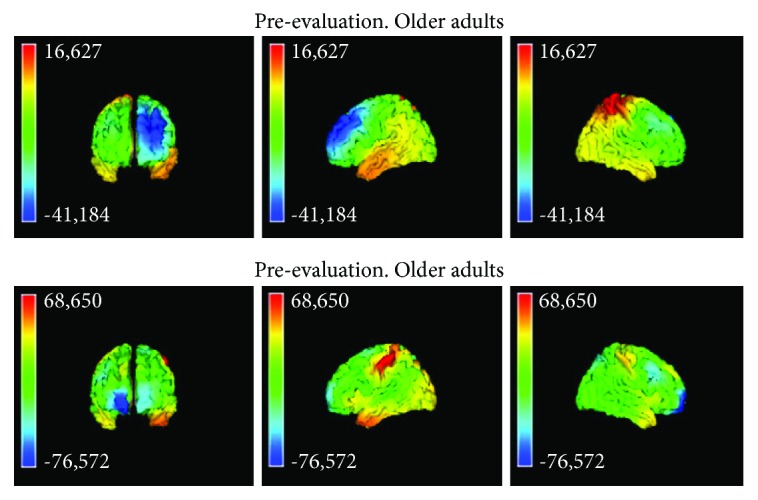
Analysis of source localization in basal and posttraining evaluation, within age groups.

In older adults, the activity in the basal evaluation was observed in the left superior and middle frontal gyrus, right parietal superior lobe, and right postcentral gyrus. In the group of older adults, the activity in the postevaluation was observed in the left postcentral gyrus and in the right lateral orbitofrontal gyrus (Figure 9).

## 5. Discussion

To the best of our knowledge, this is the first cerebral study assessing a reasoning training task in younger and older adults. The results confirm the effects of cognitive training on the brain and behavior, since the posttraining evaluation showed less brain activity and a better performance in the proposed cognitive task in both younger and older adults. On the other hand, the results confirmed hypothesis (a) of greater activation in younger adults during the basal evaluation compared to older adults. However, the results do not confirm hypothesis (b) since there were no increases in brain activity in older adults after training. In addition, hypothesis (c) on the effects of training on psychological variables is partially verified. The number of correct answers increased in the posttraining tasks; at the same time, reaction times grew unexpectedly. Globally considered cerebral results show that older adults may have less efficient cerebral resources for cognitive processing. Posttraining performance among older subjects is comparatively poor with respect to younger subjects, evidencing older adults' reduced cognitive capacity to buffer high demands. In addition, the trend of lower brain activation and worse performance in older adults, in both the baseline task and posttraining, may be interpreted as an age-dependent phenomenon rather than as a result of the demand of the task.

Through the psychological behavior of the participants, it is observed that both the number of successes and the reaction times increased after training in the two age groups. However, after training, the number of correct answers in younger adults reached almost 75% compared to the number of correct answers of the older ones that reached a little more than 50%. These results are consistent with an extensive body of information that supports a greater neurobiological decline that accompanies aging and explains why older adults obtain worse results than younger adults in cognitive performance tests [13, 29]. However, aging studies reveal large interindividual differences in cognitive performance where older adults' cognitive performance may equal that of younger adults with training and practice [30]. In this sense, the ACTIVE longitudinal study shows that older subjects who receive cognitive training maintain an improved capacity to reason above the baseline for ten years [31].

The increased reaction times after training, specifically in the younger group, contradict the hypothesis stated in the present study, which was based on the usual assumption that training an ability implies the diminution of the time required for its processing. However, when the peculiarities of the type of trained reasoning are taken into account, the increase in processing time is an index of robust cerebral consequences of training. The reasoning process applied by subjects after training is a computational step-by-step procedure which is fully explicit and compositional. That is, subjects must first process the cards composing the items, then combine them and apply the definition of a set. This is a well-known recursive or computational task [32] which consumes time as a linear function of its logical and relational complexity. In neuroscientific literature, there are only some initiatory studies on neural realizations of compositional processes in the brain [33, 34].

Therefore, while, in the untrained (basal) evaluation, the subject induced which rules could be applied without clues, in the trained version, the subject computed or applied deductive rules in a recursive and compositional way. Trained subjects did not need to grope for heuristic shortcuts as they had to in the basal evaluation. In this case, increased time is consequently an evidence of the effect of training.

The results in the source localization show a greater cerebral activation in the basal evaluation (which is more demanding) in both age groups. Bilateral cooperation is present in the realization of the basal evaluation, but the activation in the group of younger adults is located in the frontal areas, while in the group of older adults, the activation affects posterior as well as anterior areas (see Figures 8 and 9). In this case, the reviewed literature [35] attributes this kind of overrecruitment to a compensatory activation that allows a task to be successfully carried out. It is important to note that in the basal evaluation, the subject must discover the rules that fit the elements of each item. Therefore, the basal evaluation involves a greater use of cerebral and cognitive resources than if the instructions of the task are already known. In the case of older adults, both perceptive resources and more complex abstract resources are used in the basal evaluation, which contradicts the results of the reviewed literature [36]. In particular, some investigations found that due to the loss of sensory acuity, there was a decrease in the activation of anterior areas in favor of the activation of posterior areas [10, 37]. This loss means that basic cognitive operations and familiar tasks become more complex for older adults, and they have to relearn new modulations of brain activity and cognitive resources. However, the fact that the task of the study is visual explains in part why there is brain activity in the anterior areas.

The localization of sources of brain activity after training in younger adults goes from being bilateral to focal. In particular, activation is now focused on the left medial angular gyrus, the temporal and frontal areas, and the left superior frontal area. Other investigations also related these cerebral areas to deductive reasoning [38]. Therefore, the results show that the estimate of the demand of the task posttraining in this group has decreased with respect to the baseline task. In the case of older adults, the training does not cause such remarkable brain activity changes, and the strategy of bilateral activation of anterior and posterior areas is maintained. In particular, the parietal and fronto-orbital areas are activated, which again produces a wide overlap between the perceptual and abstract resources also used in the basal evaluation. The surprising lower brain activation posttraining with respect to the basal evaluation contradicts hypothesis (b) about older adults. One explanation could be a ceiling effect linked to high levels of demand in older adults [1]. That is, when older adults face a demanding task that is beyond their capacity, their performance level and brain activity both decrease [13]. However, the better cognitive results and the fact that the activation in the two groups is lower after training are proofs of the effect of training on cognitive and cerebral activity.

In summary, bilaterality may be a marker of how the brain adapts to maintain cognitive function in demanding tasks in both age groups. However, the differential bilateral locations across age groups indicate that the tendency of the brain to modulate is determined by age. One limitation of the study concerns the fact that all tasks are visual, thus restricting the possibility of verifying if posterior activation in older adults is due to the visual tasks. The high demand of the tasks and the short training period of the study may explain why older subjects improved 20% less than younger adults. Future research should replicate these results with nonvisual tasks and longer training periods to profoundly understand the benefits obtained from practice and training at a neurological level.

## Figures and Tables

**Table 1:** Repeated measures analysis of variance for the number of correct and incorrect responses preevaluation and postevaluation in younger and older adults.

Effect	Repeated measures analysis of variance with effect sizes and powers (data_saveMeanxTask_repeated measures) Sigma-restricted parameterization Effective hypothesis decomposition				
	SS	Dgr. of freedom	MS	*F*	*p*
Intercept	395255.1	1	395255.1	2105.690	0.001
Groups	4539.9	1	4539.9	24.186	0.001
Error	15767.5	84	187.7		
Correct vs. incorrect	47302.5	1	47302.5	188.596	0.001
Correct vs. incorrect ∗ groups	3680.4	1	3680.4	14.674	0.001
Error	21068.3	84	250.8		

**Table 2:** Repeated measures analysis of variance for the reaction time preevaluation and postevaluation in younger and older adults.

Effect	Repeated measures analysis of variance with effect sizes and powers (data_saveMeanxTask_repeated measures) Sigma-restricted parameterization Effective hypothesis decomposition				
	SS	Dgr. of freedom	MS	*F*	*p*
Intercept	885743564	1	885743564	921.6854	0.001
Groups	1633924	1	1633924	1.7002	0.019
Error	80724353	84	961004		
TR	15492871	1	15492871	40.9207	0.327
TR ∗ groups	7727593	1	7727593	20.4106	0.195
Error	31802969	84	378607		

## Data Availability

The data used to support the findings of this study are available from the corresponding author upon request.
